# Temporal trends in annual incidence rates for psychiatric disorders and self-harm among children and adolescents in the UK, 2003–2018

**DOI:** 10.1186/s12888-021-03235-w

**Published:** 2021-05-03

**Authors:** Lukasz Cybulski, Darren M. Ashcroft, Matthew J. Carr, Shruti Garg, Carolyn A. Chew-Graham, Nav Kapur, Roger T. Webb

**Affiliations:** 1 Centre for Mental Health & Safety, Division of Psychology & Mental Health, School of Health Sciences, Faculty of Biology, Medicine, and Health, The University of Manchester and Manchester Academic Health Sciences Centre, Manchester, M13 9PL UK; 2 NIHR Greater Manchester Patient Safety Translational Research Centre, School of Health Sciences, Faculty of Biology, Medicine and Health, The University of Manchester, Manchester Academic Health Science Centre, Oxford Road, Manchester, M13 9PL UK; 3 Centre for Pharmacoepidemiology and Drug Safety, Division of Pharmacy and Optometry, School of Health Sciences, Faculty of Biology, Medicine and Health, The University of Manchester, Manchester, UK; 4 Neuroscience & Experimental Psychology, Manchester Academic Health Science Centre, University of Manchester and Royal Manchester Children’s Hospital, Central Manchester University Hospitals NHS Foundation, Manchester, UK; 5 School of Medicine, Faculty of Medicine and Health Sciences, Keele University, Staffs, ST5 5BG UK; 6 Greater Manchester Mental Health NHS Foundation Trust, Manchester, UK

## Abstract

**Background:**

There has been growing concern in the UK over recent years that a perceived mental health crisis is affecting children and adolescents, although published epidemiological evidence is limited.

**Methods:**

Two population-based UK primary care cohorts were delineated in the Aurum and GOLD datasets of the Clinical Practice Research Datalink (CPRD). We included data from 9,133,246 individuals aged 1–20 who contributed 117,682,651 person-years of observation time. Sex- and age-stratified annual incidence rates were estimated for attention-deficit/hyperactivity disorder (ADHD) and autism spectrum disorder (ASD) (age groups: 1–5, 6–9, 10–12, 13–16, 17–19), depression, anxiety disorders (6–9, 10–12, 13–16, 17–19), eating disorders and self-harm (10–12, 13–16, 17–19) during 2003–2018. We fitted negative binomial regressions to estimate incidence rate ratios (IRRs) to examine change in incidence between the first (2003) and final year (2018) year of observation and to examine sex-specific incidence.

**Results:**

The results indicated that the overall incidence has increased substantially in both boys and girls in between 2003 and 2018 for anxiety disorders (IRR 3.51 95% CI 3.18–3.89), depression (2.37; 2.03–2.77), ASD (2.36; 1.72–3.26), ADHD (2.3; 1.73–3.25), and self-harm (2.25; 1.82–2.79). The incidence for eating disorders also increased (IRR 1.3 95% CI 1.06–1.61), but less sharply. The incidence of anxiety disorders, depression, self-harm and eating disorders was in absolute terms higher in girls, whereas the opposite was true for the incidence of ADHD and ASD, which were higher among boys. The largest relative increases in incidence were observed for neurodevelopmental disorders, particularly among girls diagnosed with ADHD or ASD. However, in absolute terms, the incidence was much higher for depression and anxiety disorders.

**Conclusion:**

The number of young people seeking help for psychological distress appears to have increased in recent years. Changes to diagnostic criteria, reduced stigma, and increased awareness may partly explain our results, but we cannot rule out true increases in incidence occurring in the population. Whatever the explanation, the marked rise in demand for healthcare services means that it may be more challenging for affected young people to promptly access the care and support that they need.

**Supplementary Information:**

The online version contains supplementary material available at 10.1186/s12888-021-03235-w.

## Introduction

Mental illness constitutes one of the leading causes of disability in the United Kingdom (UK) [1]. They are associated with several adverse trajectories, including unemployment, low income, homelessness, criminality, being assaulted or bullied, self-harm, suicide and other causes of premature death [2]. It is known that psychiatric disorder in adulthood often begins in childhood and adolescence [3]; it has been estimated that three-quarters of individuals who experience mental illness during adulthood will have met diagnostic criteria for a psychiatric disorder before reaching their 18th birthdays [4]. Thus, early intervention, prevention and treatment are important to enhance long-term outcomes. In the UK, there has been a shift in the public discourse relating to mental illness, with psychological health and wellbeing during childhood and adolescence now at the forefront of the public health agenda. In 2016 the Mental Health Taskforce of NHS England published the report ‘The Five Year Forward View for Mental Health’, which emphasised children and young people as being a “priority group for mental health promotion and prevention” [5] The UK Department of Health has also provided an additional £1.4 billion for Child and Adolescent Mental Health Services (CAMHS) between 2015 and 2020 [6] .

Studies conducted in high-income countries indicate marked increases in numbers of children and adolescents diagnosed with neurodevelopmental disorders and mental illnesses during recent decades [7]. Despite growing public concern that more children and adolescents in the UK may be affected by these conditions, there are no comprehensive and up-to-date published data on annual trends in the incidence rates in this demographic group. The most recent evidence reporting on the incidence of attention deficit/hyperactivity disorder (ADHD) [8] and depression [9, 10] indicate a stable trend or slight increases over time up until 2013. During the same period incidence of eating disorders appears to have remained stable in younger adolescents, but decreased in older adolescents [11]. Evidence for temporal trends in incidence of anxiety disorders [12] and autism spectrum disorder (ASD) [13] is even more dated, describing trends in rates up until the year 2011. These studies also indicate a stable trend in incidence over time. Investigation of the incidence of self-harm, a behaviour that is strongly linked to poor mental health, has demonstrated an increase among adolescents in the UK [14, 15] in recent years.

In addition to being somewhat dated, individual studies have drawn data from different populations and settings and have varying cohort inclusion criteria. They also differ in terms of diagnostic classification and follow-up duration and period, hindering inter-study comparison. To tackle these between-condition comparability issues and to address the gap in the evidence base, we conducted a comprehensive population-based investigation that examined temporal trends in the incidence of neurodevelopmental disorders, mental illnesses, and self-harm among young people in a single study cohort for the first time. This enabled us to assess age- and gender-specific temporal trends in incidence for multiple conditions with a uniquely high degree of comparability, as well as make use of the accurate routine primary and secondary care linkages that are available in the Clinical Practice Research Datalink (CPRD).

## Methods

### Data source

We delineated two cohorts utilising data from two datasets in the Clinical Practice Research Datalink (CPRD): GOLD and Aurum [16]. Because the two datasets are equivalent, we present in this study estimates that were created by combining data from both datasets. Aurum is a database of individuals registered with general practices in England. It consists of anonymised patient records that contain information about diagnoses, prescribed medication, and referrals to secondary healthcare services. It can also be linked to the Index of Multiple Deprivation (IMD), which combines socioeconomic indicators to create a composite ecological measure of deprivation based on practice location [17]. Clinical data are captured in the CPRD using Read codes [18]. The Aurum dataset covers approximately 13% of England’s population, containing over 7 million anonymised patient records from 731 practices, and is considered to be broadly nationally representative in terms of sex, age, and ethnicity [16]. We used the July 2019 release, which contained 7,125,786 active patient records. GOLD is also a primary care database that contains essentially equivalent information to that held in Aurum. It draws data from over 600 general practices in the UK that use an electronic patient record system that is different from that used by Aurum”. We used a “bridging” file to identify practices that have migrated from the GOLD dataset to the Aurum dataset and removed those practices from the GOLD dataset. This ensured that the study dataset contained no duplicate patient records.

### Study design

#### Cohort members

We applied the same inclusion criteria to both Aurum and GOLD datasets (Supplementary materials Fig. S1 and S2). Children and adolescents aged 1–19 during the study’s observation period, 1st January 2003 to 31st December 2018, entered the cohorts if they had been registered with their practice for at least 12 months before entry. Cohort members were followed up until the earliest of: index self-harm episode or diagnosis of mental illness, death, date of transfer out of practice, last date of data collection, study end date or their 20th birthday. We chose this age cut-off as this is when adolescence ends according to the World Health Organization’s (WHO) definition and to facilitate comparison with other recently published research [8, 14, 19].

### Study measures

#### Psychiatric diagnoses and self-harm

We classified incident diagnosed cases and self-harm episodes using lists of Read codes that were generated by exhaustive searching of the CPRD’s medical directory [20]. We identified the earliest recorded occurrence of a psychiatric disorder or non-fatal self-harm episode for each individual in the study cohort and noted the calendar year in which these events occurred. Incidence estimates from each dataset were then combined to create a single set of estimates. The following diagnostic categories were examined because they are the most common psychiatric illnesses in childhood and adolescence: anxiety disorders, ADHD, autism, depression, and eating disorders [21]. We also chose to examine self-harm because of its strong association with poor mental health and since recent evidence from the UK has shown incidence increases in recent years [14]. The search terms and the resulting code lists were then reviewed and refined by three experienced clinical academics in our team (a psychiatrist with expertise in self-harm and suicide: NK; an academic GP with expertise in mental health: CCG; and a child & adolescent psychiatrist: SG). Because the descriptors for some Read codes can seem ambiguous, we categorised codes as being either ‘stringent’ or ‘inclusive’ according to their perceived likelihood of correctly ascertaining a relevant diagnosis or self-harm episode. This approach was taken because recent evidence has demonstrated a decrease in the number of recorded depression diagnoses and an increase in the number of related symptoms that are recorded [22]. In the UK GPs are incentivised to comply with review and coding protocols stipulated by the Quality and Outcomes Framework (QOF) if they record a diagnosis of ‘depression’ using Read codes. There is evidence that some practitioners may engage in ‘strategic labelling’ (using a code such as ‘low mood’ that does not trigger the need for follow up coding) to circumvent financial penalties [23]. To account for these phenomena, we present estimates for all mental illnesses that included individuals with both symptom and diagnostic Read codes. Incidence rates that include cases captured by diagnostic codes only are presented separately in the supplementary materials. For a full list of all Read codes included in each diagnostic category, please see the supplementary materials.

#### Deprivation

We measured deprivation through the Index of Multiple Deprivation (IMD). The IMD provides a composite measure of deprivation based on information regarding income, employment, crime, barriers to housing and services, and health and living environment. Scores are provided for small geographical areas, known as Lower-layer Super Output Area (LSOA), which correspond to roughly 1500 individuals. IMD scores at LSOA level are then assigned according to individual patient’s postcodes, and placed in quintiles, with a higher score indicating higher levels of deprivation [17].

### Statistical analyses

We calculated annual incidence rates for each condition stratified by sex and by age group (1–5, 6–9, 10–12, 13–16 & 17–19 years). We generated these estimates by manually counting the number of incident cases in each stratum in each year and then dividing them by the person-time at risk in the corresponding stratum. Finally, these stratified rates were directly standardised for IMD quintile and age group. For the 1–5 years age group we only estimated incidence rates for ADHD and autism, as diagnosis of the other conditions examined is exceptionally rare at this young age. For the same reason, we did not estimate incidence rates for self-harm or eating disorders among children aged below 10 years. We modelled the count data by fitting negative binomial models. We examined temporal changes in the overall annual incidence rates between the first and final year of the study by generating incidence rate ratios (IRRs), adjusted for deprivation, sex, and age group. We also estimated IRRs stratified by sex and age group, adjusted for deprivation, to examine how change might vary across different age and gender groups. We also tested for gender interactions in models that were adjusted for deprivation quintile, year and age. Finally, we modelled the predicted annual changes to incidence after adjusting for deprivation and sex. Statistical analyses were completed using STATA 16.

## Results

### Cohort members

In the Aurum dataset, we assessed 23,840,626 unique patent records for eligibility. Of these, we included data from 4,566,623 individuals that contributed 114,601,017 person-years of observation. We assessed 19,959,796 records for eligibility in the GOLD dataset, and included 2,418,680 for analysis, contributing 28,867,639 person-years to the denominator. Please see supplementary materials (Fig. S1 and S2) for a more detailed graphical illustration of entry into and exit out of the study cohort.

### Mental illnesses

**Fig. 1:**
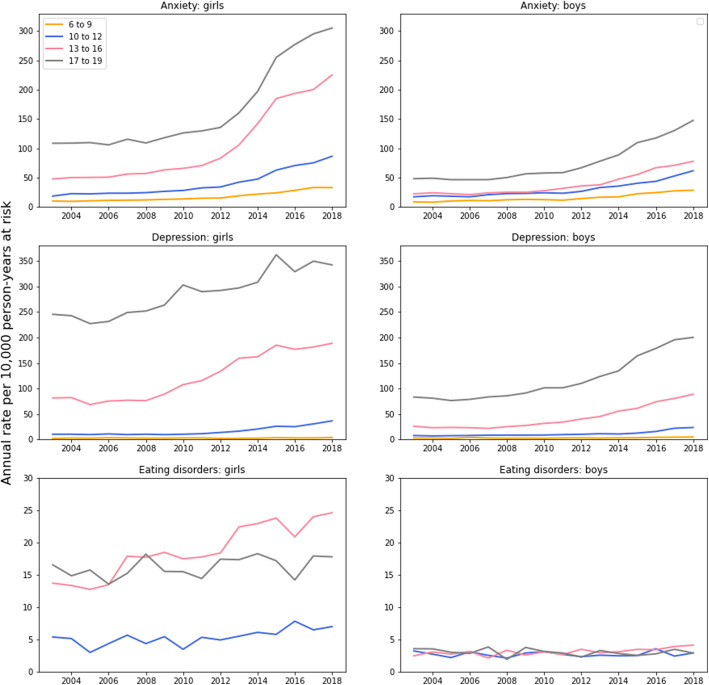
Temporal trends in annual incidence rates for mental illnesses by age group, 2003–2018

Depression and anxiety disorders were the most commonly diagnosed mental illnesses followed by eating disorders (Table 1 and Fig. 1). The incidence of depression and anxiety disorders increased in all age and groups over time for both sexes, whereas for eating disorders, increases occurred in girls of all ages and boys aged 13–16 (Table 1, Fig. 1 and supplementary Fig. S3). Incidence rate ratios measuring overall change in incidence rates between 2003 and 2018, adjusted for age, sex, and deprivation quintile, demonstrated increases in the incidence of depression (IRR 2.4; 95% 2.03, 2.77), anxiety disorders (IRR 3.5; 95% CI 3.18, 3.89), and eating disorders (IRR 1.3; 95% CI 1.06, 1.61). The highest incidence of depression was observed in the 17–19 age group, in both girls (343 per 10,000 person years in 2018; 95% CI 332–354) and boys (200 in 2018, 95% CI 192–208). After adjusting for year, age, and deprivation, we observed an effect of sex (IRR 2.13 95% CI 2.04–2.23). Rates of similar magnitude were observed for anxiety disorders in 2018 in the 17–19 age group (girls: 305; 95% CI 295–316 boys: 148; 95% CI 141–155). As can be seen in Table 1, the relative increase over time was higher in the 10–12 and 13–16 age groups for both depression and anxiety disorders. Formal testing showed that the sex-specific IRRs varied significantly according to age in all conditions examined and in both sexes, except for anxiety disorders among boys; for the latter category, there was no statistically significant evidence of heterogeneity in change in incidence between the first and last observation years across age groups (*p* = 0.267) (Supplementary materials Table S2). For anxiety disorders, girls aged 10–12 and 13–16 saw particularly sharp increases over time, with incidence rate ratios of 4.6 (95% CI 4.0–5.3), and 4.6 (95% CI 4.1–5.1) observed, respectively. As with depression, incidence rates were higher in girls than boys (IRR 1.79 95% CI 1.72–1.84). The incidence of eating disorders was much lower compared to depression and anxiety disorders. The highest incidence rate observed for this diagnostic category was during 2018 at ages 13–16 years in girls: 25 per 10,000; 95% CI 22–27. The incidence was much higher in girls than boys: IRR 4.43 (95% CI 4.13–4.76). Please see the supplementary materials for a complete array of annual incidence rates presented by sex, age group, and condition, and formal tests for effect modification by age and gender (Supplementary materials Table S1, S2, and S3).

**Table 1:** Deprivation adjusted incidence rate ratios for mental illnesses between first and final year of the study’s observation period by age group and gender

	Aged 6–9		Aged 10–12		Aged 13–16		Aged 17–19	
	2003	2018	2003	2018	2003	2018	2003	2018
Female								
Anxiety								
Cases, n	153	624	209	1170	680	3626	1035	3158
Incidence rate	10	33	19	87	48	225	109	305
Incidence rate ratio	3.2		4.6		4.6		2.6	
95% CI)	(2.6, 3.9)		(4.0, 5.3)		(4.1, 5.1)		(2.3, 3.0)	
Depression								
Cases, n	28	69	113	500	1155	3107	2274	3548
Incidence rate	1	4	10	36	81	189	245	343
Incidence rate ratio	1.9		3.6		2.1		1.3	
(95% CI)	(1.2, 3.0)		(2.9, 4.4)		(1.9, 2.5)		(1.2, 1.5)	
Eating disorders								
Cases, n			60	96	196	416	161	204
Incidence rate			5	7	14	25	17	18
Incidence rate ratio			1.3		1.6		1.0	
(95% CI)			(0.9, 1.8)		(1.2, 2.1)		(0.8, 1.3)	
Male								
Anxiety								
Cases, n	149	565	228	878	380	1350	560	1797
Incidence rate	9	29	18	62	23	78	48	148
Incidence rate ratio	3.2		3.5		3.4		2.8	
(95% CI)	(2.7, 3.8)		(2.9, 4.2)		(3.0, 3.9)		(2.3, 3.3)	
Depression								
Cases, n	46	92	100	336	439	1553	961	2428
Incidence rate	3	5	8	23	26	89	83	200
Incidence rate ratio	1.7		3.0		3.4		2.4	
(95% CI)	(1.2, 2.4)		(2.4, 3.8)		(2.9, 3.8)		(2.2, 2.6)	
Eating disorders								
Cases, n			42	42	42	73	42	37
Incidence rate			3	3	2	4	3	3
Incidence rate ratio			0.8		1.7		0.9	
(95% CI)			(0.5, 1.4)		(1.2, 2.4)		(0.6, 1.4)	

Incidence rates are rounded to closest interger

### Neurodevelopmental disorders

**Fig. 2:**
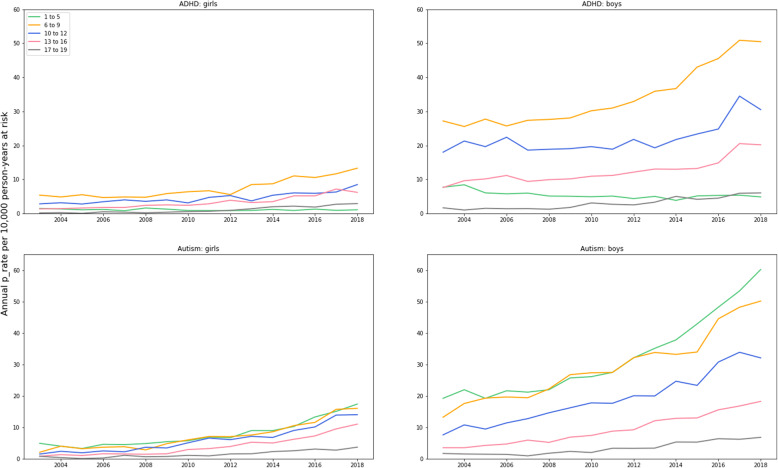
Temporal trends in annual incidence rates for neurodevelopmental disorders by age group, 2003–2018

The incidence of ASD increased over time in all age groups (IRR 2.36 95% CI 1.72–3.26). It was most commonly diagnosed in the 1–5 age group (Table 2, Fig. 2 and supplementary Fig. S4), with rates being highest in 2018 for both boys (60; 95% CI 57–64) and girls (17; 95% CI 16–19). In terms of relative increases among boys, the IRRs tended to be comparable or slightly higher than those seen for anxiety disorders and depression (Table 2). For girls the relative increases were more substantial, with IRRs of 7.5 (95% CI 5.1–11.1; 6–9 age group), 9.2 (95% CI 5.6–15.1; 10–12 age group), and 13.0 (95% CI 7.1–23.9; 13–16 age group). Overall, the incidence of ADHD increased between 2003 and 2018 (IRR 2.36 95% CI 1.73–3.25). Our stratified analyses showed that this increase occurred in all age groups across both sexes, except for the 1–5-year-olds, among whom the incidence rate decreased over time, with age group being a statistically significant effect modifier. In 2018, the highest age-specific incidence rates for ADHD were among 6–9-year-olds for both sexes (boys: 50; 95% CI 47–54; girls: 13; 95% CI 12–15). For both conditions, rates were higher in boys than in girls (Autism: IRR 3.53, 95%CI 3.37–3.69; ADHD: 4.35 95% CI 3.98–4.75). Please see the supplementary materials for a complete array of annual incidence rates presented by sex, age group, and condition, and formal tests for effect modification by age and gender (Supplementary materials Table S1, S2 and S3).

**Table 2:** Deprivation adjusted incidence rate ratios for neurodevelopmental disorders between first and final year of the study’s observation period by age group

	Aged 1–5		Aged 6–9		Aged 10–12		Aged 13–16		Aged 17–19	
	2003	2018	2003	2018	2003	2018	2003	2018	2003	2018
Female										
ADHD										
Cases, n	28	24	80	252	32	117	20	106	^a^	34
Incidence rate	2	1	5	13	3	9	1	6	0	3
Incidence rate ratio	0.7		2.5		3.0		4.6		14.3	
(95% CI)	(0.4, 1.3)		(1.9, 3.2)		(2.0, 4.2)		(2.8, 7.4)		(3.4, 59.7)	
Autism										
Cases, n	90	372	31	302	17	192	12	187	8	43
Incidence rate	5	17	2	16	2	14	1	11	1	4
Incidence rate ratio	3.5		7.5		9.2		13.0		4.6	
(95% CI)	(2.8, 4.5)		(5.1, 11.1)		(5.6, 15.1)		(7.1, 23.9)		(2.2, 9.8)	
Male										
ADHD										
Cases, n	153	110	454	987	233	431	129	351	20	76
Incidence rate	8	5	27	50	18	31	8	20	2	6
Incidence rate ratio	0.6		1.9		1.7		2.6		3.5	
(95% CI)	(0.5, 0.8)		(1.6, 2.2)		(1.4, 2.0)		(2.1, 3.3)		(2.1, 5.7)	
Autism										
Cases, n	379	1334	221	964	99	448	60	315	20	85
Incidence rate	20	60	13	50	8	33	4	18	2	7
Incidence rate ratio	3.2		3.8		4.5		5.1		4.0	
(95% CI)	(2.7, 3.8)		(3.2, 4.4)		(3.3, 5.3)		(3.7, 7.1)		(2.5, 6.5)	

Incidence rates are per 10,000 person years and were directly standardised for Index of Multiple Deprivation (IMD) quintile. Incidence rates are rounded to closest interger.

^a^ The CPRD stipulates that cell counts below 5 are masked to protect confidentiality

### Self-harm

**Fig. 3:**
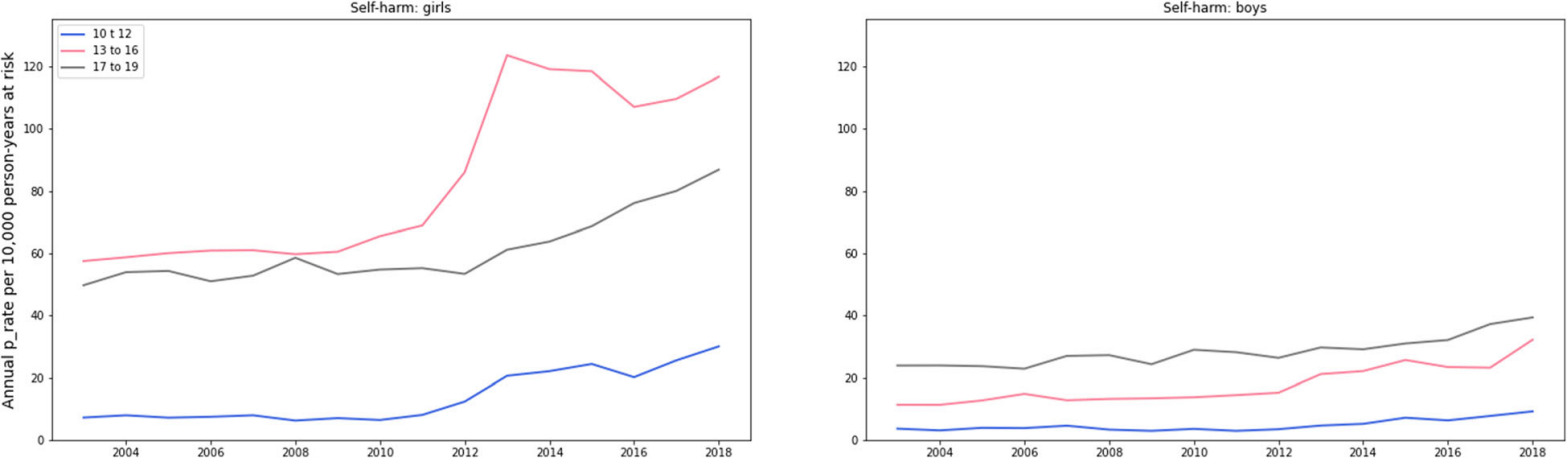
Temporal trends in annual incidence rates for self-harm by age group, 2003–2018

Self-harm incidence rates increased across both sexes and all age groups between 2003 and 2018 (IRR 2.25 95% CI 1.82–2.79: supplementary materials Fig. S5). As with mental illnesses, the incidence rates were generally highest at the end of the observation period, and the rates of some age groups seemed to increase more steeply after 2011 (Table 3 and Fig. 3). The rate was the highest among girls in 2018 aged 13–16 (117; 95% CI 111–122) followed by girls aged 17–19 (87; 95% CI 81–92). Across all age groups, incidence was considerably higher in girls than in boys (IRR 3.39 95% CI 3.17–3.62). The relative increase over time ranged from IRR 1.7 (95% CI 1.4–1.9) among 17–19-year-old boys to 4.2 (95% CI 3.3–5.3) among girls aged 10–12; IRRs varied significantly according to age. Please see the supplementary materials for a complete array of annual incidence rates presented by sex, age group, and condition, and formal tests for effect modification by age and gender (Supplementary materials Table S1, S2 and S3).

**Table 3:** Deprivation adjusted incidence rate ratios for self-harm between first and final year of study’s observation period by age group and by gender

	Aged 10–12		Aged 13–16		Aged 17–19	
	2003	2018	2003	2018	2003	2018
Female						
Cases, n	81	413	817	1934	475	952
Incidence rate	7	30	58	117	50	87
Incidence rate ratio	4.2		1.9		1.8	
(95% CI)	(3.3, 5.3)		(1.7, 2.2)		(1.6, 2.0)	
Male						
Cases, n	48	133	193	569	276	496
Incidence rate	4	9	11	33	24	39
Incidence rate ratio	2.5		2.9		1.7	
(95% CI)	(1.8, 3.6)		(2.4, 3.4)		(1.4, 1.9)	

Incidence rates are per 10,000 person years and were directly standardised for Index of Multiple Deprivation (IMD) quintile. Incidence rates are rounded to closest interger

### Sensitivity analyses: stringent vs. inclusive coding lists and GOLD vs. Aurum datasets

Results generated using our ‘stringent’ coding lists revealed similar temporal trends for all diagnostic categories examined other than depression and anxiety disorders. We observed an overall decrease over time in the incidence of depression, and a much less substantial increase over time for anxiety disorders. Please see the supplementary material (Fig. S6) for a side-by-side comparison of results generated with the “stringent” coding list.

## Discussion

In the absence of comprehensive up-to-date population-based epidemiological evidence, we examined temporal trends in the annual incidence of neurodevelopmental disorders, mental illnesses and self-harm in young people. Between 2003 and 2018 incidence rates for ADHD, anxiety disorders, autism, depression, and self-harm increased in both sexes. In some age groups the incidence more than doubled, with increases being particularly pronounced during the later years of the study’s observation period.

The temporal increases in incidence rates that we report for depression are, in terms of their magnitude, consistent with those reported from previous studies conducted in the UK [22] and in Norway [24]. In both sexes, incidence was higher at ages 13–16 and 17–19, but younger age groups saw larger relative increases. The increases in incidence of anxiety disorders followed a similar pattern, with the highest incidence recorded in the two oldest age groups, mirroring results reported from studies conducted in Wales [12] and Sweden [25]. The increases in incidence for depression and anxiety disorders that we observed were primarily accounted for by a growth in the number of individuals with recorded symptoms, rather than diagnostic codes, which too is consistent with evidence from the UK up until 2015 [22]. Studies examining prescription rates of antidepressants also show large relative increases in recent years in Sweden [26] and the UK [22]. A recently conducted study based on CPRD GOLD investigating eating disorder incidence between 2004 and 2014 reported a stable trend for girls aged 11–15 [11]. In our analysis, incidence rates nearly doubled in girls aged 13–16, with more modest increases in the 10–12 and 17–19 age groups. The increases in self-harm were of a similar magnitude to those observed for affective disorders. Girls aged 13–16 had the highest incidence, but rates increased at a faster rate in in the 10–12 group. Evidence from England & Wales report increases of a similar magnitude among adolescent girls aged 13–16 [14] and 10–14 [15] only, whereas we observed increases in both sexes and all age groups.

There is evidence for increases in prevalence of known risk factors for mental illnesses and self-harm. For example, recent data have demonstrated an increase in prevalence of maternal depression in the UK [27], a well-established risk factor for anxiety disorder and depression among children and adolescents. Individuals who live in more socially deprived areas are more likely to have mental illnesses; deprivation levels in England have since 2004 increased in households with adolescents and young adults [28]. There is also a mounting body of evidence for a dose-response relationship between social media usage and poor mental health outcomes in young people [21, 29]. Greater social media use has been linked to online harassment, sleep deficits, low self-esteem and poor body image, which are associated with an increased risk for developing depression, anxiety disorders and eating disorders [29]. Recent evidence indicates that suicide among 15–19-year-olds in England and Wales has increased between 2009 and 2017 [30]. Whilst increased exposure to risk factors may explain some of the increases that we report for mental illnesses and self-harm, it is likely that we have largely uncovered previously unidentified need.

Compared to the other conditions examined, we observed a greater variety in the magnitude of temporal change in incidence of autism between boys and girls and specific age groups. The relative increase was particularly pronounced in females, with IRRs of 8.2, 10.2, and 20.5 in girls aged 6–9, 10–12, and 13–16, respectively. It is important to note that incidence rates among these groups were very low at the beginning of the study period. Thus, while the increases in relative terms are substantial, they are small in absolute terms. Autism incidence was highest among 1–5-year-old boys, which is slightly lower than previously reported in the UK [31]. In terms of temporal change, studies from Sweden [32, 33] and Denmark [32] have reported prevalence increases in diagnosis that are of a comparable magnitude to the temporal trends that we observed. ADHD incidence increased in all age groups, except for among 1–5-year-olds, among whom rates decreased slightly over time in both sexes. Across most age groups studied, our findings were similar to those reported from a recent UK study showing stable incidence rates between 2003 and 2011, with an upwards trend in 2011–2013. Our results may be a continuation of this trend. Studies from the past decade show that the number of diagnoses and prescriptions of methylphenidate have increased by similar magnitudes in Denmark [32, 34], Sweden [32, 35] and the UK [36]. Increases in parental age, in-utero exposure to antidepressants, or extremely low birth weight have been proposed as explanations for the increased incidence of ADHD and autism [7], although raised prevalence of rare risk factors, or of those that are not strongly associated with the outcome, is unlikely to account for the large increases in incidence that we and other studies have observed. Moreover, both ADHD and autism have a strong genetic component, with an estimated heritability of 70–80% [37, 38]. For these reasons, there is a growing consensus that increases in the incidence of neurodevelopmental illnesses observed over the last 20 years are mostly due to factors independent of aetiology, such as increased levels of awareness, detection, diagnosis and availability of services [7].

Considerable caution is needed in making inferences regarding causal mechanisms from temporal trends in incidence rates at population level because these estimates to an unknown extent are influenced by factors that are independent of underlying disease aetiology. For example, increases over time likely reflect changes to ascertainment, service provision and treatment. Changes to diagnostic criteria may influence rates of diagnosis. However, clinicians in the UK primarily rely on the International Classification of Disease (ICD) system for diagnosis, which during the duration of our study has remained unchanged. Reductions to stigma associated with mental illnesses and increased awareness among teachers and parents may also contribute to increased help seeking. Estimates from studies that are less likely to be influenced by these factors report more modest increases in incidence over time. For example, results from the UK Child Adolescent Mental Health Survey conducted in 1999, 2004 and 2017, in which several thousand randomly sampled children and adolescents were assessed for mental illnesses and exposure to their risk factors, reported increases in the prevalence of mental illnesses among individuals aged 5–15 ranging from 9.7 to 11.2%. Moreover, whilst the proportion of self−/parent-reported mental illnesses has increased in the UK, the degree of reported psychological distress has remained stable [39].

A potential limitation of our study is that we could not determine the validity or relative severity of diagnosed conditions and self-harm episodes. However, as several studies have demonstrated, there is a significant risk of under-ascertainment by adopting a case definition that is unduly stringent [9, 22]. We invite interested readers to consider the supplementary files for a side-by-side comparison of results generated using case definitions of varying levels of sensitivity (Fig. S4). Moreover, because the CPRD does not comprehensively capture detailed contextual information pertaining to neurodevelopmental disorders, mental illnesses and self-harm, we are unable to determine if the increases in incidence reflect true changes to underlying levels of psychological distress and psychopathology in the population. Whilst we implemented a 12-month clearance period to reduce the risk of including prevalent cases, it is possible that some prevalent cases were included as the CPRD does not have complete coverage across the entire study period. Our results reflect the incidence of recorded diagnoses of mental illnesses in primary care; cases recorded in other healthcare settings may therefore be missing. Finally, this study used data prior to the COVID-19 pandemic and resulting restrictions and change in service delivery.

## Conclusions

In this study, we show that the number of recorded mental illness and self-harm-related primary care episodes have increased substantially among children and adolescents in the UK in recent years. These findings are consistent with evidence of increased demand for specialist mental health services [40] and raise several important questions for researchers, commissioners, and policymakers. Secular trends of mental illness-related presentations in healthcare settings are influenced by a complex interaction of countervailing forces, many of which are independent of disease aetiology. Future research is needed to determine if increased demand for treatment reflects a real deterioration in psychological wellbeing in the population and, if so, to identify the underlying causes as this may lead to more targeted interventions. This could partly be accomplished by more frequent updates to the UK Child Adolescent Mental Health Survey, which provides prevalence estimates of psychiatric illnesses and their risk factors that to a lesser extent are influenced by factors unrelated to underlying morbidity. Identifying the most effective responses to the reported increase in demand represents a major challenge for policymakers and researchers alike. Whilst increasing access to treatment is a clear priority, it is evident that many of the solutions for reducing the burden of mental illnesses lie beyond child and adolescent mental health services, and preventive efforts will need to address underlying determinants, such as poverty, bullying, educational stressors, and parental mental illness. Because most psychiatric illnesses begin before adulthood, there is consensus among clinicians that preventative efforts targeting young people can lead to greater personal, social and economic benefit than interventions delivered at later stages of life [41]. The ongoing public health response in the UK, which stresses the need for parental support and outlines plans for increased collaboration between schools and mental health services [6], is consistent with this view. However, evidence from meta-analyses of school-based interventions is mixed [42, 43], and more high quality evidence is needed to better understand which interventions are effective for whom and in what settings, and which factors are associated with their successful implementation.

### Added value of this study

Using the UK Clinical Practice Research Link (CPRD) Aurum and GOLD datasets, we delineated a cohort study based on routinely collected primary care patient electronic health records. We combined the study cohorts and investigated temporal trends in the incidence of anxiety disorders, autism, attention-deficit hyperactivity disorder (ADHD), depression, eating disorders, and non-fatal self-harm in children and adolescents aged 1–19 years during 2003–2018. By presenting temporal trends in incidence rates of self-harm and several neurodevelopmental disorder and mental illness diagnostic categories in the same study cohort, we have addressed some of the key limitations that are inherent with inter-study comparisons, providing an up-to-date overview that hitherto has been lacking from the evidence base. Results indicate that an increasing number of children and adolescents are seeking help for neurodevelopmental disorders, mental illnesses and self-harm behaviour. For several age groups and diagnostic categories the incidence rose by two-fold or more, with especially steep increases occurring toward the end of the study’s observation period.

## Supplementary Information

**Additional file 1: Fig. S1.** Flow of cohort members in CPRD Aurum cohort. **Fig. S2.** Flow of cohort members in CPRD GOLD cohort.

**Additional file 2.** Supplementary materials.

**Additional file 3: Fig. S3.** Observed annual and predicted incidence rates of anxiety disorders, depression and eating disorders through a linear time trend. **Fig. S4.** Observed annual and predicted incidence rates of attention-deficit hyperactivity disorder (ADHD) and autism spectrum disorder (ASD) through a linear time trend. **Fig. S5.** Observed annual and predicted rates of self-harm through a linear time trend.

**Additional file 4: Fig. S6.** Annual incidence rates: inclusive vs. stringent estimate.

**Additional file 5: Table S1.**

**Additional file 6: Table S2.** Tests for effect modification by age and gender for incidence rate ratios indicating change in incidence between 2003 and 2018. **Table S3.** Tests for effect modification by gender for incidence rate ratios measure indicating change in incidence between 2003 and 2018.

## Data Availability

Clinical Practice Research Datalink (CPRD) study datasets cannot be shared publicly due to licencing restrictions. Researchers wishing to conduct their own investigations using CPRD data should contact the Knowledge Centre directly at the following email address: enquiries*@*cprd*.*com. The STATA code that was used to generate the results is available on request.
