# Supplementary material for: Temporal trends in annual incidence rates for psychiatric disorders and self-harm among children and adolescents in the UK, 2003–2018
Source: BMC Psychiatry. 2021 May 3;21:229. doi: 10.1186/s12888-021-03235-w (PMC8092997; doi:10.1186/s12888-021-03235-w)
Supplement: Supplementary file 1 — Additional file 1: Fig. S1. Flow of cohort members in CPRD Aurum cohort. Fig. S2. Flow of cohort members in CPRD GOLD cohort. [file 12888_2021_3235_MOESM1_ESM.docx]

**Temporal trends in annual incidence rates for psychiatric disorders and self-harm among children and adolescents in the UK, 2003-2018**

**Authors:**
Lukasz Cybulski^1,2^ (Corresponding author: lukeznder@gmail.com)
Darren M. Ashcroft^2,3^
Matthew J. Carr^2,3^
Shruti Garg, University of Manchester^5^
Carolyn A. Chew-Graham^4^

Nav Kapur^1,2,6^
Roger T. Webb^1,2^

**Organisational affiliations**

1 Centre for Mental Health & Safety, Division of Psychology & Mental Health, School of Health Sciences, Faculty of Biology, Medicine, and Health, The University of Manchester and Manchester Academic Health Sciences Centre, Manchester, M13 9PL, UK

2 NIHR Greater Manchester Patient Safety Translational Research Centre, School of Health Sciences, Faculty of Biology, Medicine and Health, The University of Manchester, Manchester Academic Health Science Centre, Oxford Road, Manchester, M13 9PL, UK

3 Centre for Pharmacoepidemiology and Drug Safety, Division of Pharmacy and Optometry, School of Health Sciences, Faculty of Biology, Medicine and Health, The University of Manchester, Manchester, United Kingdom

4 School of Medicine, Faculty of Medicine and Health Sciences, Keele University, Staffs, UK ST5 5BG

5 Neuroscience & Experimental Psychology, Manchester Academic Health Science Centre, University of Manchester and Royal Manchester Children's Hospital, Central Manchester University Hospitals NHS Foundation, Manchester, UK

6 Greater Manchester Mental Health NHS Foundation Trust

**Fig S1**. Flow of cohort members in CPRD Aurum cohort

Patients in August 2018 version of CPRD *Aurum* database ^(1)^

(*n* = 29,178,979)

Without records acceptable for research purposes ^(2)^

(*n* = 5,338,353)

With records acceptable for research purposes

(*n* = 23,840,626)

Not registered and/or eligible during study period ^(3,4)^

(*n* = 19,274,003)

Registered and eligible for at least 1 day during study period (*n =* 4,566,623)

Incident psychiatric disorder or self-harm episodes

(*n* = 282,362 )

4,566,623

1. Permanent registrations only
2. Acceptability defined by CPRD as meeting certain quality standards.
3. Study period: 1st January 2003 to 31 December 2018.
4. Reasons: a) 20^th^ birthday occurred before beginning of study period, b) left practice before beginning of study period, or c) incident psychiatric episode occurred prior to study start.

**Fig S2**. Flow of cohort members in CPRD GOLD cohort

Patients in August 2018 version of CPRD *GOLD* database ^(1)^

(*n* = 19,959,796)

Without records acceptable for research purposes ^(2)^

(*n* = 2,496,254)

With records acceptable for research purposes

(*n* = 17,463,542)

Removed because practices migrated from GOLD to Aurum dataset ^(3,4)^

(*n* =4,563,419;

165 practices)

With records acceptable for research purposes after taking into account migration from GOLD to Aurum dataset

(*n =* 13,433,362)

Not registered and/or eligible during study period ^(3,4)^

(*n* =11,014,682)

Registered and eligible for at least 1 day during study period (*n =* 2,418,680)

Incident psychiatric disorder or self-harm episodes

(*n* = 70,678)

)

1. Permanent registrations only
2. Acceptability defined by CPRD as meeting certain quality standards.
3. Study period: 1st January 2003 to 31 December 2018.
4. Reasons: a) 20^th^ birthday occurred before beginning of study period, b) left practice before beginning of study period, or c) incident psychiatric episode occurred prior to study start.
