# Supplementary material for: Temporal trends in annual incidence rates for psychiatric disorders and self-harm among children and adolescents in the UK, 2003–2018
Source: BMC Psychiatry. 2021 May 3;21:229. doi: 10.1186/s12888-021-03235-w (PMC8092997; doi:10.1186/s12888-021-03235-w)

**Temporal trends in annual incidence rates for psychiatric disorders and self-harm among children and adolescents in the UK, 2003-2018**

**Authors:**
Lukasz Cybulski^1,2^ (Corresponding author: lukeznder@gmail.com)
Darren M. Ashcroft^2,3^
Matthew J. Carr^2,3^
Shruti Garg, University of Manchester^5^
Carolyn A. Chew-Graham^4^

Nav Kapur^1,2,6^
Roger T. Webb^1,2^

**Organisational affiliations**

1 Centre for Mental Health & Safety, Division of Psychology & Mental Health, School of Health Sciences, Faculty of Biology, Medicine, and Health, The University of Manchester and Manchester Academic Health Sciences Centre, Manchester, M13 9PL, UK

2 NIHR Greater Manchester Patient Safety Translational Research Centre, School of Health Sciences, Faculty of Biology, Medicine and Health, The University of Manchester, Manchester Academic Health Science Centre, Oxford Road, Manchester, M13 9PL, UK

3 Centre for Pharmacoepidemiology and Drug Safety, Division of Pharmacy and Optometry, School of Health Sciences, Faculty of Biology, Medicine and Health, The University of Manchester, Manchester, United Kingdom

4 School of Medicine, Faculty of Medicine and Health Sciences, Keele University, Staffs, UK ST5 5BG

5 Neuroscience & Experimental Psychology, Manchester Academic Health Science Centre, University of Manchester and Royal Manchester Children's Hospital, Central Manchester University Hospitals NHS Foundation, Manchester, UK

6 Greater Manchester Mental Health NHS Foundation Trust

**Fig S3**. Observed annual and predicted incidence rates of anxiety disorders, depression and eating disorders through a linear time trend.


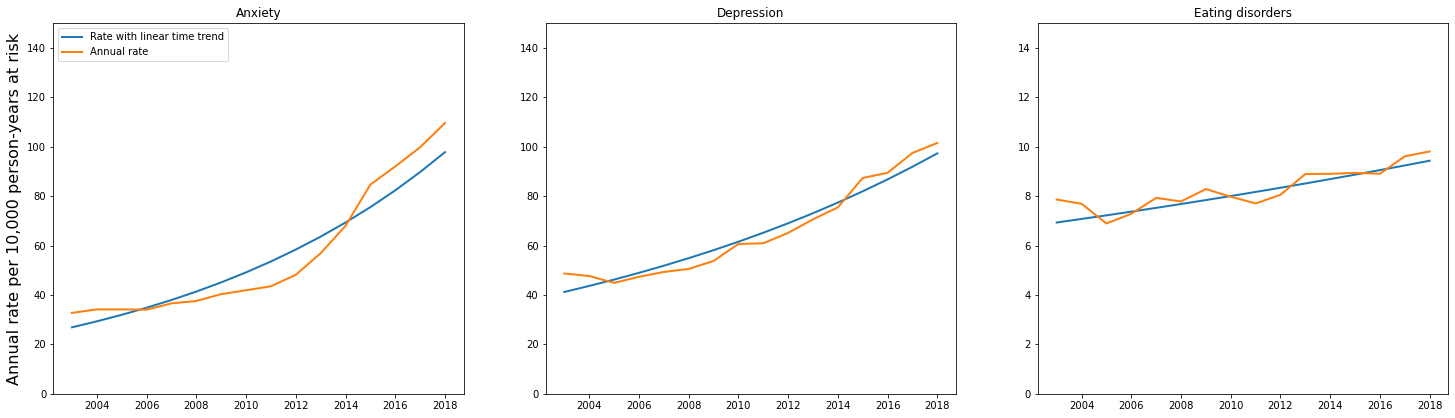


**Fig S4**. Observed annual and predicted incidence rates of attention-deficit hyperactivity disorder (ADHD) and autism spectrum disorder (ASD) through a linear time trend.


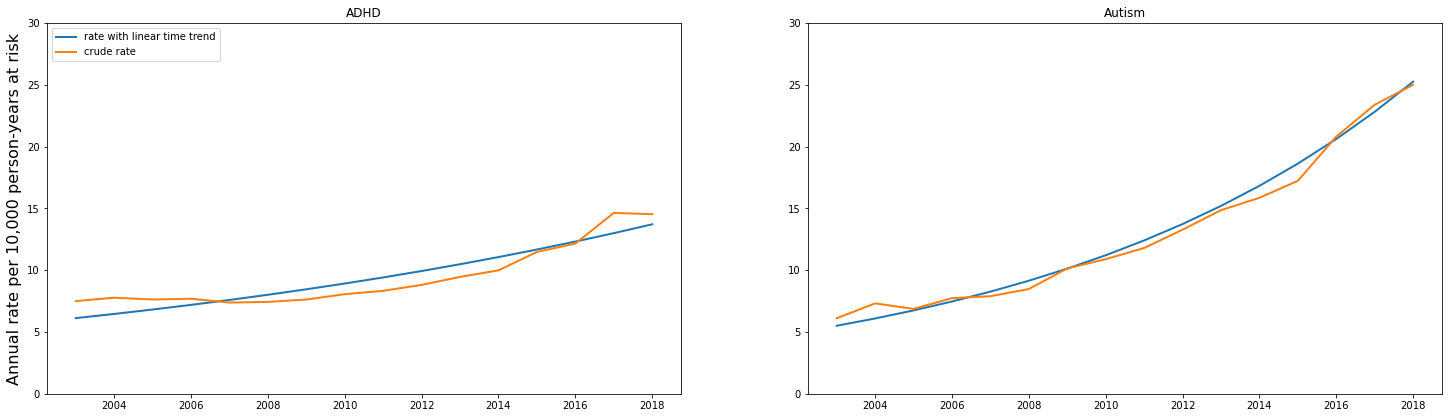


**Fig S5**. Observed annual and predicted rates of self-harm through a linear time trend.


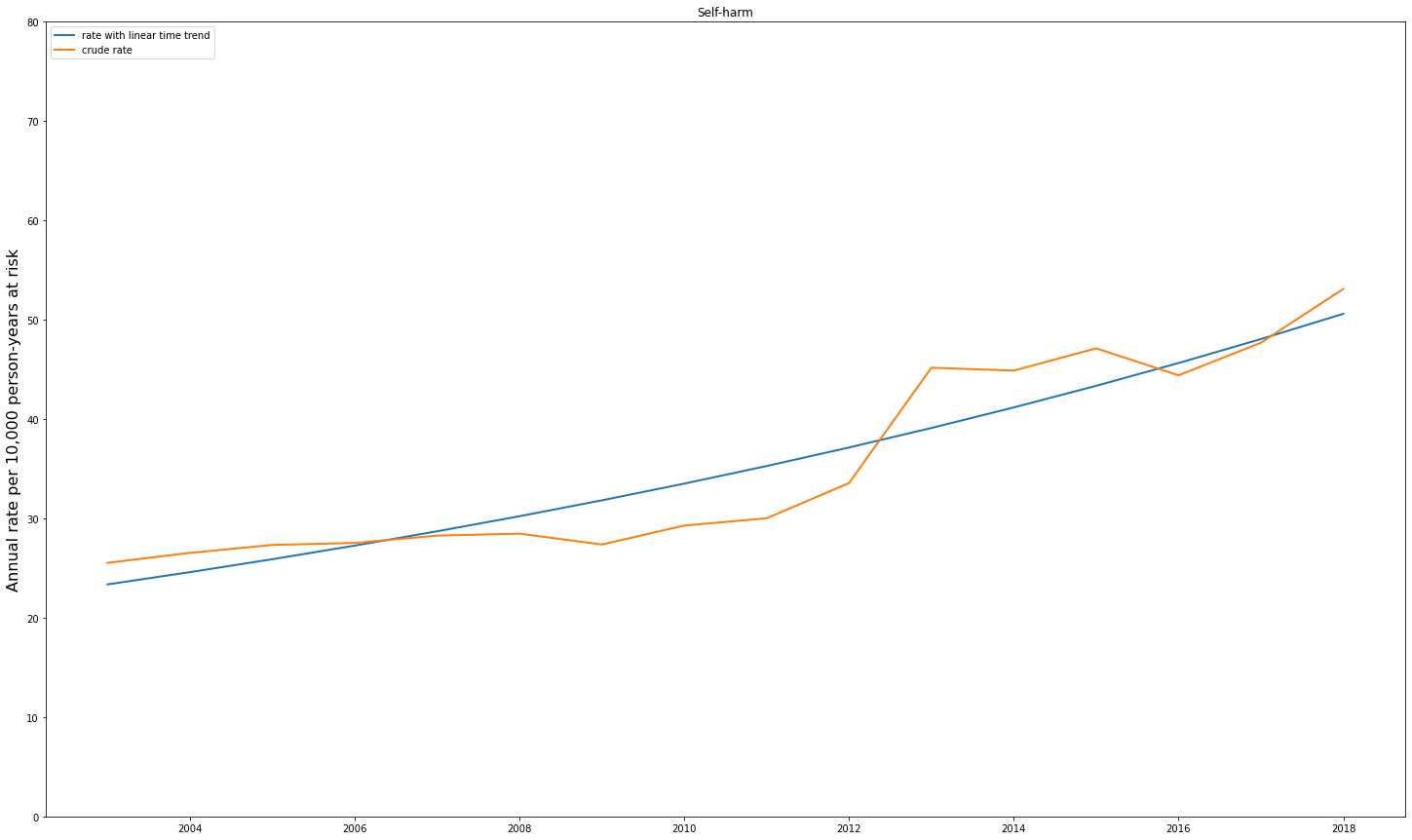

Supplement: Supplementary file 3 — Additional file 3: Fig. S3. Observed annual and predicted incidence rates of anxiety disorders, depression and eating disorders through a linear time trend. Fig. S4. Observed annual and predicted incidence rates of attention-deficit hyperactivity disorder (ADHD) and autism spectrum disorder (ASD) through a linear time trend. Fig. S5. Observed annual and predicted rates of self-harm through a linear time trend. [file 12888_2021_3235_MOESM3_ESM.docx]
