# Supplementary material for: Temporal trends in annual incidence rates for psychiatric disorders and self-harm among children and adolescents in the UK, 2003–2018
Source: BMC Psychiatry. 2021 May 3;21:229. doi: 10.1186/s12888-021-03235-w (PMC8092997; doi:10.1186/s12888-021-03235-w)
Supplement: Supplementary file 4 — Additional file 4: Fig. S6. Annual incidence rates: inclusive vs. stringent estimate. [file 12888_2021_3235_MOESM4_ESM.docx]

**Temporal trends in annual incidence rates for psychiatric disorders and self-harm among children and adolescents in the UK, 2003-2018**

**Authors:**
Lukasz Cybulski^1,2^ (Corresponding author: lukeznder@gmail.com)
Darren M. Ashcroft^2,3^
Matthew J. Carr^2,3^
Shruti Garg, University of Manchester^5^
Carolyn A. Chew-Graham^4^

Nav Kapur^1,2,6^
Roger T. Webb^1,2^

**Organisational affiliations**

1 Centre for Mental Health & Safety, Division of Psychology & Mental Health, School of Health Sciences, Faculty of Biology, Medicine, and Health, The University of Manchester and Manchester Academic Health Sciences Centre, Manchester, M13 9PL, UK

2 NIHR Greater Manchester Patient Safety Translational Research Centre, School of Health Sciences, Faculty of Biology, Medicine and Health, The University of Manchester, Manchester Academic Health Science Centre, Oxford Road, Manchester, M13 9PL, UK

3 Centre for Pharmacoepidemiology and Drug Safety, Division of Pharmacy and Optometry, School of Health Sciences, Faculty of Biology, Medicine and Health, The University of Manchester, Manchester, United Kingdom

4 School of Medicine, Faculty of Medicine and Health Sciences, Keele University, Staffs, UK ST5 5BG

5 Neuroscience & Experimental Psychology, Manchester Academic Health Science Centre, University of Manchester and Royal Manchester Children's Hospital, Central Manchester University Hospitals NHS Foundation, Manchester, UK

6 Greater Manchester Mental Health NHS Foundation Trust

**Supplementary Fig S6**. Annual incidence rates: inclusive vs. stringent estimate.


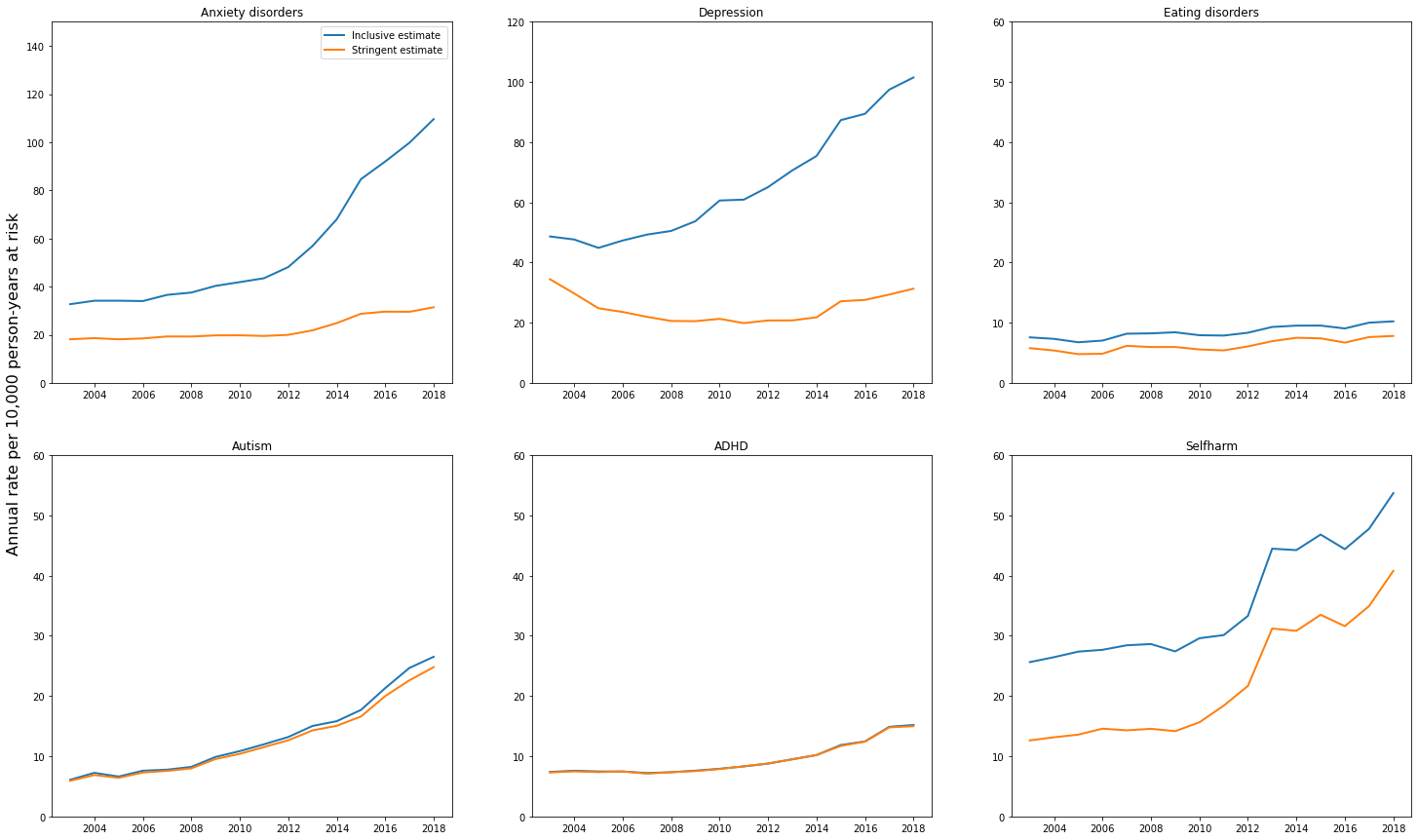


Note. We used Read codes to identify episodes of self-har and psychiatric disorders. Because the descriptors for some Read codes can seem ambiguous, we categorised codes as being either ‘stringent’ or ‘inclusive’ according to their perceived likelihood of correctly ascertaining a relevant diagnosis or self-harm episode.
