# Supplementary material for: Temporal trends in annual incidence rates for psychiatric disorders and self-harm among children and adolescents in the UK, 2003–2018
Source: BMC Psychiatry. 2021 May 3;21:229. doi: 10.1186/s12888-021-03235-w (PMC8092997; doi:10.1186/s12888-021-03235-w)
Supplement: Supplementary file 6 — Additional file 6: Table S2. Tests for effect modification by age and gender for incidence rate ratios indicating change in incidence between 2003 and 2018. Table S3. Tests for effect modification by gender for incidence rate ratios measure indicating change in incidence between 2003 and 2018. [file 12888_2021_3235_MOESM6_ESM.docx]

| **Table S2.** Tests for effect modification by age and gender for incidence rate ratios indicating change in incidence between 2003 and 2018 | | | | | | |
| --- | --- | --- | --- | --- | --- | --- |
|  | **Incidence rate ratios by age group** | | | | | **P-value (effect modification by age group)** |
|  | 1-5 | 6-9 | 10-12 | 13-16 | 17-19 |  |
| **Female** |  |  |  |  |  |  |
| Anxiety disorders | - | 3.2 | 4.6 | 4.7 | 2.8 | < 0.001 |
| Depression | - | 1.9 | 3.6 | 2.2 | 1.3 | < 0.001 |
| Eating disorders | - | - | 1.3 | 1.8 | 1.1 | 0.02 |
| ADHD | 0.7 | 2.5 | 3.0 | 4.5 | 14.5 | < 0.001 |
| Autism | 3.5 | 7.7 | 9.3 | 13.0 | 4.6 | < 0.001 |
| Self-harm | - | - | 4.1 | 2.0 | 1.7 | < 0.001 |
| **Male** |  |  |  |  |  |  |
| Anxiety disorders | - | 3.2 | 3.5 | 3.4 | 2.9 | 0.27 |
| Depression | - | 1.7 | 3.0 | 3.4 | 2.4 | < 0.001 |
| Eating disorders | - | - | 0.9 | 1.7 | 0.8 | 0.03 |
| ADHD | 0.6 | 1.9 | 1.7 | 2.6 | 3.5 | < 0.001 |
| Autism | 3.2 | 3.8 | 4.2 | 5.1 | 4.0 | 0.04 |
| Self-harm | - | - | 2.5 | 2.9 | 1.7 | < 0.001 |

| **Table S3**. Tests for effect modification by gender for incidence rate ratios measure indicating change in incidence between 2003 and 2018. | | | |
| --- | --- | --- | --- |
|  | **Incidence rate ratio by sex** | |  |
|  | Female | Male | **P-value (effect modification by sex)** |
| **Anxiety disorders** |  |  |  |
| 6-9 | 3.2 | 3.2 | 0.90 |
| 10-12 | 4.6 | 3.5 | 0.02 |
| 13-16 | 4.7 | 3.4 | < 0.001 |
| 17-19 | 2.8 | 2.9 | 0.28 |
| **Depression** |  |  |  |
| 6-9 | 1.9 | 1.7 | 0.68 |
| 10-12 | 3.6 | 3.0 | 0.29 |
| 13-16 | 2.2 | 3.4 | < 0.001 |
| 17-19 | 1.3 | 2.4 | < 0.001 |
| **Eating disorders** |  |  |  |
| 10-12 | 1.3 | 0.9 | 0.19 |
| 13-16 | 1.8 | 1.7 | 0.93 |
| 17-19 | 1.1 | 0.8 | 0.27 |
| **ADHD** |  |  |  |
| 1-5 | 0.7 | 0.6 | 0.90 |
| 6-9 | 2.5 | 1.9 | 0.06 |
| 10-12 | 3.0 | 1.7 | 0.007 |
| 13-16 | 4.5 | 2.6 | 0.04 |
| 17-19 | 14.5 | 3.5 | 0.03 |
| **Autism** |  |  |  |
| 1-5 | 3.5 | 3.2 | 0.48 |
| 6-9 | 7.7 | 3.8 | 0.002 |
| 10-12 | 9.3 | 4.2 | 0.002 |
| 13-16 | 13 | 5.1 | 0.002 |
| 17-19 | 4.6 | 4.0 | 0.78 |
| **Self-harm** |  |  |  |
| 10-12 | 4.1 | 2.5 | 0.02 |
| 13-16 | 2.0 | 2.9 | < 0.001 |
| 17-19 | 1.7 | 1.7 | 0.52 |
